# Editorial: Bioengineering of biomimetic microenvironments for cardiac tissue engineering

**DOI:** 10.3389/fbioe.2023.1339120

**Published:** 2023-12-08

**Authors:** Elisabetta Rosellini, Aldo R. Boccaccini, Federico Quaini, Yu Shrike Zhang

**Affiliations:** ^1^ Department of Civil and Industrial Engineering, University of Pisa, Pisa, Italy; ^2^ Department of Materials Science and Engineering, Institute of Biomaterials, Friedrich-Alexander-University (FAU), Erlangen, Germany; ^3^ Department of Medicine and Surgery, University of Parma, Parma, Italy; ^4^ Division of Engineering in Medicine, Department of Medicine, Brigham and Women’s Hospital and Harvard Medical School, Cambridge, MA, United States

**Keywords:** biomimetic approach, cardiac tissue engineering, scaffold, bioreactor, extracellular matrix, functionalization, signals, *in vitro* models

Congenital and acquired diseases of the heart are leading causes of morbidity and mortality worldwide. Currently available clinical treatments have limited ability to repair the damaged myocardium. Therefore, scientists and surgeons are continuously exploring new strategies to treat the injured heart. Over the past decades, cardiac tissue engineering emerged as a promising strategy to produce new functional cardiac tissues, oftentimes using a combination of cells, scaffolds and signals.

In consideration of the compositional and structural complexity of the native cardiac tissue and of the plurality of biochemical, physical, electrical and mechanical stimuli that cardiac cells physiologically experience *in vivo*, the generation of biomimetic culture environments, including both scaffolds and bioreactors, can play an important role in advancing the efforts toward the achievement of native heart tissue-like constructs.

The scope of this Research Topic is to provide an overview of the current state of the art concerning different strategies to obtain biomimetic scaffolds and bioreactors for cardiac tissue engineering. Overall, the Research Topic comprises 4 peer-reviewed manuscripts, among which 2 are original articles and 2 are reviews.

Biomimetic scaffolds are structures that mimic nature. They are designed and developed taking inspiration from the natural scaffold of native tissues, that is the extracellular matrix. It is well-known that the extracellular matrix not only provides structural support to tissues, but also offers a pattern for spatial organization, cellular binding sites and signaling molecules, both under physiological and pathological conditions. In the article published by Spedicati et al., the authors described the preparation and characterization of biomimetic scaffolds, for applications in the development of 2D and 3D models of early-stage post-infarct human cardiac fibrosis, to be used as predictive platforms for the preclinical validation of new therapies. A bioartificial combination of poly(caprolactone), poly(3,4-dihydroxyphenylalanine) and gelatin was used to fabricate 2D scaffolds by electrospinning and 3D scaffolds by 3D printing. The novelty of the work arises from scaffold design, which mimicked the different stages of myocardial fibrosis, in terms of composition, thickness, stiffness and structure. Morphological, physicochemical and biological characterizations were performed. In particular, human cardiac fibroblast cultures, of both atrial and ventricular origins, were evaluated and results showed cell adhesion, proliferation, differentiation toward myofibroblast phenotype and deposition of pathological cardiac extracellular matrix. Overall, these findings pointed out the suitability of the developed platforms to model both atrial and ventricular cardiac fibrosis, exploitable for future preclinical testing of new strategies for cardiac regeneration.

The development of biomimetic microenvironments for cell culture implies not only the appropriate design of scaffolds mimicking the native extracellular matrix, but also the assembly of biomimetic bioreactors providing physical stimuli, similar to those experienced by cells in the *in vivo* dynamic context. In particular, the delivery of physiological-like electrical and/or mechanical stimuli is critical for the maturation of a functional cardiac tissue. In this regard, Gabetti et al. reported the development of a versatile electrical stimulator, for cardiac tissue engineering applications. While commercial stimulators are burdened by restrained waveform modulation, high cost and bulkiness, and currently available customized solutions allow limited parameter tunability, the reported compact stimulator combines low cost and the possibility to simultaneously test different electrical stimulation patterns on multiple samples. Specifically, finite element analysis was adopted to test the distribution of the electric field and current density within the culture chamber. Importantly, results were further validated by means of experimental measurements. Reliability and accuracy of the device were also assessed through performance tests. Moreover, biological investigations on neonatal rat cardiac cells were carried out, comparing the effects of monophasic and biphasic pulsed electrical stimulation. Thus, the conditions of electrical stimulation able to enhance electrical functionality and promote synchronous contraction were identified.

Moving to the two review papers published in the article Research Topic, the aim was to address different relevant aspects implicated in the development of biomimetic environments in myocardial tissue engineering. In particular, the manuscript by Bernava and Lop, starting from a biological description of the native heart tissue, offers a deep insight on the design and fabrication of biomimetic scaffolds for cardiac tissue engineering, able to emulate the cardiac extracellular matrix. Moreover, the authors provided an overview of the current preclinical and clinical applications of engineered cardiac tissues, taking in consideration three different tissue engineering approaches: the implantation of unseeded scaffolds, the transplantation of scaffold-free healthy cells and the hybrid treatments based on the combination of cells and scaffolds. Finally, the current state of the art concerning the application of tissue engineering in the development of *in vitro* models of the cardiac tissue was provided, documenting both the modeling of cardiac diseases, such as myocardial infarction, cardiac fibrosis and cardiac hypertrophy, and the evaluation of drug efficacy and cardiotoxicity.

In another review, Rosellini et al. focused the attention on the most advanced strategies to bioengineer scaffolds capable to mimic, in all their characteristics, the complex natural myocardial environment. In particular, the authors pointed out how the key to move cardiac tissue engineering from the research arena to routine clinical practice, could reside on the synergic combination of: i) scaffold fabrication techniques based on 3D printing/bioprinting, which allow to reproduce the complex microarchitecture of myocardial tissue; ii) bioinks based on natural biomaterials, mimicking the composition of the extracellular matrix; iii) functionalization strategies able to empower the scaffold with signals essential to guide orderly cell behaviour and tissue growth. Therefore, an overview of the state of the art on the development of biomimetic and multifunctional 3D-printed natural biomaterial-based cardiac patches was provided, additionally foreseeing their applications both in tissue repair and in the attainment of reliable *in vitro* models.

Overall, this Article Research Topic offers an updated picture on the engineering of cardiac tissue through biomimicry. Although the engineering of a functional cardiac tissue still remains an unmet ambitious goal of tissue engineering, current available literature suggests that a nature inspired design of both scaffolds and culture environment can significantly advance cardiac tissue engineering to become a new clinical therapeutic option for patients suffering from cardiac diseases, as well as a valuable instrument to develop advanced *in vitro* cardiac tissue models, for disease modelling and drug screening.

